# Discussing proton pump inhibitor deprescribing: the views of Danish GPs and older patients

**DOI:** 10.1186/s12875-020-01227-5

**Published:** 2020-08-08

**Authors:** Wade Thompson, Malene Nissen, Peter Haastrup, Jette Videbæk Le, Carina Lundby, Jesper Bo Nielsen, Dorte Ejg Jarbøl

**Affiliations:** 1 Research Unit of General Practice, University of Southern Denmark, J.B. Winsløwsvej 9, 5000 Odense, Denmark; 2 Hospital Pharmacy Funen, Odense University Hospital, Odense, Denmark; 3 OPEN, Open Patient data Explorative Network, Odense University Hospital, Odense, Denmark; 4 Research Unit of Clinical Pharmacology and Pharmacy, University of Southern Denmark, Odense, Denmark

**Keywords:** Deprescribing, Proton pump inhibitors, Shared decision-making, Communication

## Abstract

**Background:**

Deprescribing of proton pump inhibitors (PPIs) can be considered in situations where the drug may no longer be necessary; however, this requires a careful discussion between patients and healthcare providers, often general practitioners (GPs). The aim of our study was to explore how GPs discuss PPI deprescribing with patients and compare that to how older patients would like to discuss this decision.

**Methods:**

We conducted a qualitative study using semi-structured interviews with GPs (*n* = 11) and patients aged ≥65 years who were taking PPIs (*n* = 4). Analysis of interviews was based on systematic text condensation.

**Results:**

We identified four main themes: (1) Reasons PPI deprescribing comes up, (2) Considering PPI deprescribing, (3) Discussion topics, and (4) Incorporating patient preferences into PPI deprescribing decisions. We found that PPI deprescribing often comes up during consultations for other problems or due to concern about medication burden in general. GPs discussed topics related to symptom control, such as the possibility of rebound symptoms, the need to taper PPIs, and what to do if symptoms returned. This aligned with what patients felt was important to discuss. Some GPs routinely incorporated patient preferences into decisions, whereas others did not.

**Conclusion:**

When discussing PPI deprescribing, the GPs in our study generally focused on topics related to symptom control. There was variability in how and if patient preferences were discussed. Greater focus may be needed on developing mechanisms to elicit and incorporate patient preferences into PPI deprescribing decisions. Future research could also explore more systematic approaches to reassess ongoing PPI use in an effort to curb unnecessary long-term use of PPIs.

## Background

Proton pump inhibitors (PPIs) are common medications, used to treat upper gastrointestinal (GI) problems such as gastroesophageal reflux disease (GERD) and peptic ulcer disease as well as reduce risk of GI bleeding in those at elevated risk [1]. After a treatment course (typically two to 8 weeks), the need to continue the PPI should be reassessed [2, 3]. In patients whose disease/symptoms are healed and do not have a compelling indication to continue a PPI indefinitely, discontinuation of the PPI should be considered [1–3]. However, many patients eligible for possible discontinuation appear to continue PPIs beyond recommended treatment courses [4]. Deprescribing refers to the discontinuation of a medication, that is planned and supervised by a healthcare provider [5]. Consideration of PPI deprescribing may be particularly important in older persons, who are at higher risk of adverse drug events and drug interactions, and tend to use higher numbers of medications [6, 7].

The possibility of PPI deprescribing should be carefully discussed between patients and a healthcare provider [3], which will often be the patients’ general practitioner (GP). The decision should be based on a shared understanding of the benefits and harms of deprescribing compared to continuation, as well as patient values and preferences [8]. A discussion about PPI deprescribing could also include discussing the rationale for considering deprescribing, as well as the deprescribing plan [3]. A 2018 study conducted in Canada recorded and analyzed conversations surrounding PPI deprescribing in primary care, and found that much of the discussion between patients and prescribers centered on instructions for deprescribing as well as follow-up; however, this study involved prescriber training and tools on communication around PPI deprescribing [9]. As such, there has been little examination of how GPs approach PPI deprescribing discussions in routine care and whether this aligns with patient views on deprescribing discussions. Thus, the objective of our study was to examine how GPs discuss PPI deprescribing in routine practice and compare this to the views of older patients.

## Methods

We conducted face-to-face semi-structured interviews with GPs and patients. A COREQ checklist [10] is included as Additional file 1.

### Participants

GPs were recruited from February to December 2018 via personal networks from the Danish regions of Southern Denmark and Zealand. We adopted a purposeful sampling strategy applying the criteria that interviewees must have had experience discussing deprescribing of PPIs with their patients [11]. No GPs we approached refused to participate.

Patients were recruited from March to April 2019 at community pharmacies in Southern Denmark. Patients were eligible for participation if they were aged ≥65 years, had used a PPI for more than 8 weeks, were asymptomatic, and had no clear indication for long term use (i.e. did not have a history of bleeding ulcer, Barrett’s esophagus, Zollinger-Ellison syndrome, erosive esophagitis, and regular treatment with ulcerogenic drugs such as non-steroidal anti-inflammatory drugs [NSAIDs]). The indication for PPI use was gathered from participants and was not confirmed in the medical chart. Patients’ number of regularly scheduled medications was gathered from pharmacy records with the consent of the patient. Pharmacy staff did not keep track of how many patients refused to participate.

### Interviews

GP interviews were approximately 30 min, conducted in English by WT (pharmacist and PhD student with experience in qualitative research), and took place at GPs’ offices. Interviews followed an interview guide, which was developed based on existing literature surrounding shared decision-making in deprescribing, as well as barriers and enablers of deprescribing [8, 12]. The interview guide was piloted with two GPs (see Additional file 2).

Patient interviews were approximately 15 min, conducted in Danish by MN (pharmacy student), and took place at community pharmacies. These interviews also followed an interview guide (see Additional file 3), which was adapted from the GP interview guide and based on shared decision-making literature [8, 12, 13]. For both patient and GP interviews, the interviewers' pre-understanding was that shared decision-making was the optimal approach to PPI deprescribing decisions.

All interviews were audio recorded and transcribed verbatim. Transcripts and findings were not returned to participants for comments, and we did not conduct any repeat interviews. We aimed to recruit until we obtained sufficient information power [14] to achieve the objective of the study.

### Analysis

Analysis was based on systematic text condensation, which is rooted in phenomology [15]. (1) One researcher read through all interviews to determine preliminary themes and the research group met to review and agree on these preliminary themes. (2) One researcher went through interviews to identify meaning units (quotations or blocks of text that elucidate themes) and the meaning units were organized under preliminary themes from step 1. Themes were refined from the preliminary versions based on the new understanding of the data. (3) Subthemes were created under each main theme and meaning units were organized under subthemes. The meaning units from each subtheme were then synthesized into a condensate (an artificial quotation that explained the subtheme) in order to ensure the meaning units expressed the subtheme. The research team reviewed and discussed the themes, subthemes, and meaning units, and agreed upon final themes and subthemes. (4) Condensates and meaning units were used to create narrative descriptions of the content of each subtheme, which served as the results section. Quotations from patient interviews were translated from Danish into English. GP interviews were coded using Microsoft Word, and patient interviews were coded using NVivo12.

### Ethics

Interviews with patients were approved by the Danish Data Protection Agency (19/7266), while approval was not required for interviews with GPs. All patients and GPs provided written consent to participate.

## Results

We interviewed 11 GPs and 4 patients (one patient had a relative present for the interview). The characteristics of the participants are presented in Table 1. We identified four main themes (see Table 2): (1) Reasons PPI deprescribing comes up, (2) Considering PPI deprescribing, (3) Discussion topics, and (4) Incorporating patient preferences into PPI deprescribing decisions.

**Table 1:** Characteristics of participants

**GPs** (***n*** **= 11**)	
Age (mean, SD, range)	46 (7.9) [41–68]
Sex (n, %)	
Female	6 (55)
Male	5 (45)
Years practicing (mean, SD, range)	9 (7.6) [3–30]
Practice location (n, %)	
Rural	2 (18)
Urban	9 (82)
**Patients (*n*** **= 4)**	
Age (mean, SD, range)	68.5 (3.5) [65–72]
Sex (n, %)	
Female	2 (50)
Male	2 (50)
Number of regular medications (mean, SD, range)	10.5 (5.8) [5–18]

*Abbreviation*: *SD* standard deviation

**Table 2:** Summary of themes and subthemes

Theme	Subthemes	Summary
Reasons PPI deprescribing comes up	Concern about medication burden in general	PPI deprescribing often came up as part of a strategy of reducing the overall number of medication patients were taking – this concern could be brought up by patients or GPs.
	Consultations for other problems	PPI deprescribing was often tagged onto consultations for other problems rather than a reason for the consultation.
	Asking about PPI deprescribing specifically	GPs felt patients rarely brought up deprescribing of PPIs specifically; however, patients stated they might bring it up if they were worried about adverse effects.
Considering PPI deprescribing	Low-hanging fruit	GPs stated that PPIs were one of their first targets when considering deprescribing of medications.
	Searching for an indication	GPs needed to assess whether deprescribing was clinically appropriate before getting into a discussion; this could include gathering information from the patient or chart.
Discussion topics	Explaining the rationale for deprescribing	GPs stated they would talk to patients about why deprescribing was an option for them, and patients reported wanting to understand why a PPI could be deprescribed.
	Planning and follow-up	GPs stated they would talk to patients about rebound symptoms and how to manage them; patients stated they wanted to know what to do if their symptoms came back.
Incorporating patient preferences into PPI deprescribing decisions	–	GPs acknowledged that patients have different preferences related to PPI treatment; some GPs would actively discuss this and incorporate preferences into decisions whereas other GPs would not routinely talk to patients about their preferences. Patients had different views on how much they would like to discuss preferences and be involved in decisions.

Abbreviations: *GP* general practitioner, *PPI* proton pump inhibitor

### Theme 1: Reasons PPI deprescribing comes up

#### Subtheme 1: Concern about medication burden in general

GPs noted that deprescribing of PPIs could be discussed as part of a general strategy of reducing medication use in patients taking many medications. In this case, deprescribing would often be brought forward by GPs.

*“But there might be some patients who take a lot of medicine and are very old. And I say ‘how is it taking all this medicine? Would you like to get a little bit less medicine?’” (GP4).*

However, many GPs also suggested that patients could bring this up, feeling they were taking too many medications.

*“Sometimes the patients will come and say ‘I don’t want to be on all these medications. Can’t we see if we can take some of them off my list?’” (GP11).*

This was consistent with the views of patients, who also suggested that a desire to take less medication in general could lead them to ask if there was any medication that could be discontinued.

“*… is there something I could spare, because it is chemicals I put in my body, right?” (P1*).

#### Subtheme 2: Consultations for other problems

GPs said that PPI deprescribing would come up most often in consultations for other problems. PPI deprescribing would be discussed for a few minutes at the end of the consultation, for example, when the GP was ordering refills or if the GP noticed a long-term PPI as part of the medication list in the patient’s electronic health record. In these situations, GPs were primarily the ones bringing the discussion forward.

*“Actually, it’s often a part of something else. They consult me for something else and then...they want a prescription for this. And then I ask them. Do you take this regularly?” (GP8).*

*“Whenever I come across a patient who comes in for whatever reason and I see that they’ve been using proton pump inhibitors for a while, I bring up the discussion of whether they should discontinue it or not.” (GP7).*

#### Subtheme 3: Asking about PPI deprescribing specifically

GPs felt that it was rare for patients to bring up PPIs specifically.

*“With PPIs, I guess it’s mostly me. No patients come to mind asking me to help them get off the drug.” (GP5).*

However, patients discussed situations where they could bring up PPI discontinuation specifically, for example if they were worried about adverse effects or felt the medication was not effective.

*“Well, it would actually only be if I feel any side effects or if it does not work.” (P3).*

*“Well, everything is eventually cancer-causing, so if [PPIs] suddenly were shown to be cancer-causing, then I would probably ask by myself if I can get something else.” (P1).*

### Theme 2: Considering PPI deprescribing

#### Subtheme 1: Low-hanging fruit

When considering potential medication discontinuation during a medication review or during a consultation about another problem, GPs noted that PPIs were one of their first targets.

*“Because if you are doing a medication review in that frailer, old patient. You know the PPIs are one of the low hanging fruit.” (GP11).*

*“I think PPIs are one of the first things you would try to skip.” (GP9).*

Most GPs felt that PPI deprescribing was a relatively easy deprescribing discussion to have. This may have stemmed from GPs feeling the risks of PPI deprescribing were generally minimal, and that patients could always start taking the PPI again if needed.

*“And it’s a pretty easy discussion, I think. It’s a drug I consider very safe...and there are no … probably no risks associated with discontinuing the medicine. So the decision to try discontinuation is easy and straightforward.” (GP5).*

#### Subtheme 2: Searching for an indication

While GPs identified PPIs as potential low-hanging fruit, before opening a discussion about PPI deprescribing, most GPs talked about the need to assess whether PPI deprescribing could be considered in the first place. GPs stated they would attempt to identify the original indication for the PPI. This could involve finding previous endoscopy results, past consultation notes, or notes from specialists.

*“I might even before they come in... I might take a little bit of time to review the notes and when the last time they had a test [was]. What did their gastroenterologist tell them about? Should they stay on [the PPI] and for how long?” (GP11).*

*“I try to go back to find out when this problem started and how has it been treated.” (GP2).*

Most GPs stated they would also gather information from patients. For example, they would ask about ongoing symptoms, or ask patients about the history of their GI problems.

*“So normally I would ask if they have any symptoms, if they use it for any specific reason, why they are on that med in the first place.” (GP7).*

When determining whether PPI deprescribing was appropriate, GPs also spoke about assessing bleeding risk. GPs would evaluate whether a patient had a history of GI bleeding or took any medications that could increase bleeding risk, such as NSAIDs.

*“But I’m more looking into if they are taking NSAIDs or aspirin.” (GP9).*

*“If they have a risk of ulcer then I would probably let them continue. If there is something in their history about [a] bleed from the stomach or yeah if the risk is there, then I will probably leave the PPI.” (GP8).*

### Theme 3: Discussion topics

#### Subtheme 1: Explaining the rationale for deprescribing

Most GPs would explain the reason PPI deprescribing could be considered and outline how a PPI may not be necessary anymore.

*“I often say it’s not good to take any more medicine than is needed. So that’s often the rationale. And most of the patients understand it.” (GP4).*

*“Normally I tell the same story … that we gave this to you 10 years ago or 20 years ago. Your body is 10 to 20 years older. Perhaps you don’t have to [use] it anymore. Let’s see for the next 2 weeks, 3 weeks or 4 weeks, could you live without that medicine.” (GP9).*

This was consistent with patient wishes, as patients reported a desire to understand the reason PPI deprescribing was being brought up.

*“I would probably need a very good explanation why I suddenly cannot have that medicine anymore.” (P1).*

*“Well, it would be just asking why I need to quit.” (P3).*

Most GPs felt that PPIs were relatively safe, though acknowledged the rare adverse effects which have been associated with long-term PPI use. There were differing views surrounding discussing adverse effects as a reason for discontinuation. Some GPs would routinely bring this up in discussions around possible PPI deprescribing.

*“We talked about maybe changing the microorganisms in his gastrointestinal tract...that there is some newer research pointing to that being a problem.” (GP11).*

In contrast, some GPs talked about how they did not want to scare patients, in particular because deprescribing attempts may fail and the person may end up staying on the medication.

*“I try not to emphasize the risks of chronically taking the medicine because it will often end up with them having to take it, so I don’t want to scare them too much.” (GP5).*

#### Subtheme 2: Planning and follow-up

Most GPs would also discuss *how* to go about deprescribing with patients. This often included whether to taper the PPI, and how fast to taper.

*“And we also discuss how fast should they try to...taper. Some are more courageous than others...some don’t want to do anything too fast. Some others want to do it next day.” (GP7).*

Most GPs would also discuss rebound symptoms with patients. This would include explaining what rebound symptoms are.

*“Maybe your symptoms can get a little bit worse for the next 4 to 6 weeks and that’s to be expected and that’s all right. It’s not because your reflux is getting worse and worse. And I think a lot of the patients they actually tell me afterwards that they feel that that information is really useful. Because I think a lot of them do experience the rebound.” (GP11).*

Some GPs would also discuss how to manage rebound symptoms in case they occur. GPs would advise patients about using antacids or using a PPI as needed.

*“And tell them that if they are having a good night out, perhaps the next morning they would feel some stomach pain and reflux then maybe for a couple of days they could use [the PPI]. Or perhaps take some [antacid].” (GP9).*

The GP approach to discussing rebound symptom management was consistent with patient views. Patients also wanted to discuss symptom control upon discontinuation, specifically how to manage symptoms if they came back.

*“We will have to see how it goes …and if [symptoms] do come back, then I know that I can go to my physician and talk to him about it, and then he will take action.” (P1).*

Most patients understood that deprescribing would be a trial, but also wanted to be clear with their GP that they could return to taking the PPI if necessary.

*“We could try to quit because nothing will happen by trying. It is not dangerous in any way …It is only a test” (P2).*

*“But I also need to say that, okay, can we agree that now I will try this for a month … or for how long it should go on, completely without [a PPI], and then we will see how I feel.” (P1).*

GPs had differing approaches to follow-up. Most GPs would tell patients to contact them if they had any problems during the deprescribing process but would not schedule any formal follow-up.

*“I would typically tell them that you could always phone me or write me an email if you have any symptoms or concerns related to this. Just get back to me and we’ll discuss it.” (GP11).*

In contrast, some GPs scheduled follow-up appointments to reassess the deprescribing attempt.

*“So we would discuss a taper. And then make a new appointment in 4 weeks or so.” (GP7).*

Patients did not think scheduled follow-up was necessary but wanted to know they could contact their physician for help if they experienced any problems during deprescribing.

*“When I feel bad, then I would like him to help me … It should not be a regular schedule.” (P4).*

### Theme 4: Incorporating patient preferences into PPI deprescribing decisions

GPs were generally aware that patients may have different attitudes and preferences related to possible PPI deprescribing. GPs noted that some patients would be fearful to try deprescribing, whereas others may be keen, or motivated to reduce the number of pills they take. However, GPs handled preferences and attitudes towards PPIs in different ways. Some GPs were more open to incorporating patient preferences into decisions. For example, some GPs discussed continuing PPIs if patients felt strongly against deprescribing.

*“But it depends on the patient. If it’s a patient that gets very worried if we talk about discontinuation and they have a lot of other problems, PPI is sort of at the bottom of the stack.” (GP7).*

*“I’m generally open. I listen to them, I ask them, and I talk with them. I listen to what they say. And then we reach a decision that the patient is happy about and I am happy about. But I try to inform them as much as possible so they can be a part of it.” (GP8).*

*“It’s always the patient who decides. Like I just talk to them about it. And some patients would like to stop and some patients wanted to continue.” (GP4).*

Other GPs would discuss patient attitudes and preferences but may still try to persuade patients to try deprescribing.

*“I think I would try to be sure about their thoughts on it, what’s their experience … And then of course I would push them to try. I would ask them to try. At least try. But if you really make your time to explain, OK let’s try to do it in this way [reducing gradually] then I think it works out.” (GP6).*

Some GPs would not actively discuss preferences at all, feeling the patient would bring up their preferences if they felt strongly against (or in favour of) deprescribing.

*“I think it’s something about my consultation style is I believe that I’m aware of the patients’ wishes and what they’re saying. I don’t have a style of pushing something without them having a say. And I expect – the patients that I know well at least – that they know this style. So I feel that they are very comfortable with discussing their own wishes and goals.” (GP5).*

Similarly, some GPs talked about patients who would simply want the GP to make the decision without discussing the patient preferences.

*“Some of them [older patients] will just leave it up to me and tell me straight. If you think it’s a good idea, then that’s what we’ll do. There are not that many left of that kind of patient type.” (GP11).*

Patients expressed different views around their involvement in decision-making. One patient felt they would like to be the one to have the final say around the decision.

*“It would of course be the patient who has the final decision, definitely!” (P3).*

Another patient wanted to be a part of the discussion but felt that the final decision should be made by the GP.

*“Well, surely we take fifty percent part in it, don’t we? We are two people. But it is still her that needs to say yes or no … I must honestly admit that I am not more educated in that than what you can google.” (P2).*

One patient noted that if the GP asked them about discontinuing their PPI, they would just do it, as they trusted their physician.

*“Well, then I would probably just quit, because I have great confidence in what my physician tells me with regard to medicine.” (P1).*

## Discussion

### Summary of findings

Our interviews suggest PPI deprescribing discussions often come up during consultations for other problems or when there is a general aim to reduce medication burden from the GP and/or patient; however, PPI deprescribing is seldom a reason for a consultation on its own. Most of the discussions are focused on symptom control – (1) explaining a PPI may be unnecessary if there are no ongoing upper GI symptoms, (2) the possibility of rebound symptoms during discontinuation, (3) how to avoid return of symptoms during discontinuation (i.e. tapering plan), and (4) what to do if symptoms recur. This approach aligns with what is important to patients, as patients mentioned wanting to understand why a PPI could be discontinued and were concerned about the possibility of symptoms returning upon discontinuation. There were different views among GPs on the role of explicitly bringing up patient preferences during PPI deprescribing discussions, while patients had different perspectives on their role in decision-making. Many GPs saw PPIs as “low-hanging fruit” for deprescribing.

### Limitations

We recruited GPs from two regions in Denmark. Further, GPs were recruited using personal networks of the research team, and all were practicing in group practices. As such, the views and practices of the GPs in our study may not be generalizable to all Danish GPs or GPs practicing in other contexts/countries. While we instructed GPs that there were no wrong responses to questions, desirability bias among GPs is still a possible concern. Finally, we included very few patients from only one region of Denmark and our sample was younger old persons (age range 65 to 72). We hoped to recruit more patients; however, due to slow recruitment we were unable to recruit additional patients during the study period and may not have achieved sufficient information power [14]. As such, we have limited data on patient views, and the findings on patient views may not reflect the larger population of older patients taking PPIs in Denmark or abroad. However, including patients allowed us to compare the views of this PPI users to the views of GPs, and provide additional context to the views of GPs.

### Comparison with existing literature

Our findings align with the results of a recent analysis of PPI deprescribing conversations [9]. These authors also found that most of the discussion centered on planning and follow-up. While prescribers in this study did have access to training resources related to PPI deprescribing discussions before consultations, we found that the approach to discussions was similar compared to the GPs in our study. Patient views in our study are in line with what has previously been reported, with patients expressing a desire to understand the rationale for PPI deprescribing, the possibility of rebound symptoms, and what to do if symptoms return [13]. Given that GPs in our study reported discussing these topics, the PPI deprescribing practices of these GPs appeared to generally align with patient views surrounding PPI deprescribing discussions. Patients in our study were generally open to discussing deprescribing, which is consistent with a recent Australian study of PPI users [16].

The GPs in our study displayed a range of practices with respect to discussing patient preferences, suggesting that patient preferences may not always be incorporated into PPI deprescribing decisions. This finding is consistent with a Canadian study [17], which similarly found that patient preferences were not always factored into deprescribing decisions, as well as an Australian study which reported that preferences were often not incorporated into older patients’ treatment decisions [18]. We also found a range of attitudes from patients towards discussing their preferences and being part of deprescribing discussions. This is in line with an Australian interview study [19], which found older patients had different levels of desire to participate in shared-decision making around deprescribing, with some patients wanting to actively participate in shared decisions (including discussing their preferences) and other patients wanting to leave decisions up to their physician. Variability in patients’ desired level of involvement in decision-making has also been seen in a Danish context. A 2016 survey of over 6000 Danes aged 18 to 75 years found that 75% of Danes prefer to be involved in decisions about disease treatment, though 25% prefer to leave decisions up to their doctor [20].

### Implications for clinical practice and research

GPs in our study discussed many of the topics considered important to patients with respect to PPI deprescribing, which is an encouraging finding. However, there was variability in how patient preferences were discussed and incorporated into decisions. Discussing patient preferences is viewed as being a central component of shared decision-making surrounding deprescribing and attempts to help patients feel confident and satisfied with treatment decisions [8]. Our findings on how GPs discuss patient preferences suggest that shared decision-making may not always be occurring with respect to PPI deprescribing. As such, mechanisms may be needed to encourage GPs to elicit patient preferences and incorporate them into decisions on PPI deprescribing. The development and use of tools, such as patient decision aids [21] or conversation guides, may be helpful in this context. There appears to be room for improvement with respect to readability and accessibility of existing tools [22]. However, it has also been suggested that a broader approach may be needed to improve uptake of shared decision-making, for example focusing on communication skills and changing consultation models such that shared understanding of problems, and patient priorities and preferences are central to decision-making [23, 24].

Overuse of PPIs continues to be a clinical and economic concern [3, 25, 26]. GPs in our study found PPIs to be “low-hanging fruit” and relatively easy to deprescribe, which raises questions as to why PPI overuse continues to be such a problem. While GPs appeared to acknowledge long-term unnecessary PPI use as a concern, many GPs noted that PPI deprescribing was often tagged onto a consultation for another medical problem. This suggests that PPI use may not be systematically and routinely addressed and may be superseded by more pressing issues in limited available consultation time. This is consistent with a recent Danish study, which found only 25% of GPs reported having a consultation about continued PPI use in the previous year, and that few of these consultations led to changes in treatment [27]. There may be a need to explore and implement mechanisms that encourage the systematic and routine practice of assessing ongoing PPI use in general practice. Possible solutions to explore may include: involving other healthcare providers such as nurses or pharmacists [28, 29] in initiatives that address PPI use, including expected durations/reassessment dates and indications on initial PPI prescriptions, dedicated consultations for deprescribing, or setting up for systems for triaging PPI refills.

## Conclusions

When discussing PPI deprescribing, the GPs in our study typically focused on topics related to symptom control, such as the need to taper PPIs and what to do if rebound symptoms occur. These discussion topics generally aligned with what patients considered important to discuss. However, there was variability in how and if patient preferences were discussed, which may limit implementation of shared decisions surrounding PPI use. Our results suggest that greater focus may be needed on eliciting and incorporating patient preferences into PPI deprescribing decisions, including implementation of tools and models that facilitate shared decision-making. Our findings also suggest that GP discussions around PPI deprescribing are often tagged onto consultations for other problems. As such, future research could explore more systematic approaches to reassess ongoing PPI use in an effort to curb unnecessary long-term use PPIs.

## Supplementary information

**Additional file 1.** COREQ checklist

**Additional file 2.** GP interview guide

**Additional file 3.** Patient interview guide (in Danish)

## Data Availability

Transcripts from interviews are not publicly available to maintain confidentiality of participants.

## Abbreviations

GERD: Gastroesophageal reflux disease
GI: Gastrointestinal
GP: General practitioner
NSAID: Non-steroidal anti-inflammatory disease
PPI: Proton pump inhibitor
