# Supplementary material for: Discussing proton pump inhibitor deprescribing: the views of Danish GPs and older patients
Source: BMC Fam Pract. 2020 Aug 8;21:160. doi: 10.1186/s12875-020-01227-5 (PMC7415175; doi:10.1186/s12875-020-01227-5)
Supplement: Supplementary file 2 — Additional file 2. GP interview guide [file 12875_2020_1227_MOESM2_ESM.docx]

**Additional File 1: GP Interview Guide**

**Introduction**

We are conducting this study to understand how GPs discuss long-term medication use with patients. Specifically, we are interested in learning about all different aspects of GP discussions surrounding continuing a medication versus discontinuing or reducing it. The discussion will be recorded and take about 30 minutes. We would like to have a very open, informal and honest discussion, so there are no “wrong” answers or thoughts.

The interviewer will ask the GP to reflect on a situation in clinical practice where they have had to discuss whether a patient should continue taking or PPI or try to reduce/stop it.

| **TOPIC** | **PROBING QUESTIONS** | **SPECIFIC QUESTIONS** |
| --- | --- | --- |
| General thoughts | Can you recall the last time you had a discussion about continuing or discontinuing a PPI with one of your patients?  Overall, how did the discussion go? |  |
| Initiating discussions | How did the discussion come up? (i.e. did the patient bring it up or you?) | Did you go into the discussion openly or did you feel the patient should discontinue the PPI?  What options would you present to the patient? |
| Discussion points | What do you talk about when having such a consultation? |  |
| Factors important to GP | What is important to discuss with the patient for this decision? | What information is important to convey to the patient when having this discussion?  How is this information conveyed?  *e.g. benefits/harms of PPIs or benefits/harms of reducing PPI, polypharmacy* |
| Patient factors | What do patients ask or say about continuing/discontinuing their PPI? | What has been important to patients when having this discussion? |
| (Shared) decision-making | How did you end up coming to a final decision? | How do you incorporate what is important to patients into the decision (or discussion)?  How involved have patients been in coming to a final decision?  *e.g. have decisions been made by GP, by patient, or together?* |
| Practical matters | How much time have you spent having such a discussion?  What are other organizational factors that may impact the discussion? (e.g. having a nurse at your clinic, running behind that day) | How much time would you like to spend having this discussion?  If you need more time to discuss, what would you do?  Can you recall a situation where you had to postpone your discussion?  *Can present with different options* |
| Risk | Are the discussions different depending on the type of patient (e.g. older versus younger)? | How would the discussion differ?  *Can also probe about different risk factors* |
| Preparedness | Are there situations where you would need time to gather more information before having the discussion (e.g. review guidelines, gather more clinical information about patient)? |  |
| Planning | How did you talk to the patient about the plan going forward? | *E.g. tapering plan, follow-up* |
| Additional thoughts | Are there any other factors which are important to you when discussing continuation versus discontinuation of a PPI? |  |
