# Supplementary material for: Discussing proton pump inhibitor deprescribing: the views of Danish GPs and older patients
Source: BMC Fam Pract. 2020 Aug 8;21:160. doi: 10.1186/s12875-020-01227-5 (PMC7415175; doi:10.1186/s12875-020-01227-5)
Supplement: Supplementary file 3 — Additional file 3. Patient interview guide (in Danish) [file 12875_2020_1227_MOESM3_ESM.docx]

# **Additional File 2: Patient Interview Guide**

Erstat ”PPI” med respondentens præparatnavn (Pantoprazol, Omeprazol, Lanzoprazol, Esomeprazol, Rabeprazol) eller det ord, som respondenten bruger for sin PPI (fx syrehæmmende tablet)

Start interviewet med:

- Hvor gammel er du?
- Hvor længe har du taget PPI?
- Ved du, hvorfor du tager PPI? (Hvad er indikationen? - for at udelukke Barrett’s, tidligere blødning eller NSAID-brug)

Derefter følg denne guide:

| **Emne** | **Sonderende spørgsmål** | **Specifikke spørgsmål** |
| --- | --- | --- |
| Generelle tanker | Har du før haft diskuteret med din læge, om du skulle stoppe med PPI? | Hvis ja, hvad snakkede I om?  Hvis nej, hvad ville du tænke hvis din læge sagde at det var muligt at stoppe med PPI eller nedsætte dosis? |
| Indledende diskussioner | Hvad vil kunne få dig til at overveje selv at tage diskussionen op med din læge? | Skal der være bivirkninger ved medicinen? Manglende effekt? Du tager for mange tabletter? |
| Diskussionspunkter | Hvad tænker du er vigtigt at diskutere med din læge i forbindelse med om du skulle stoppe PPI? | Hvilke informationer kunne du godt tænke dig?  Har du brug for at vide:   - hvorfor du kan stoppe med den? (rationalet bag, gevinster) - Hvad bivirkninger ved langtidsbrug kan være? - Hvilke symptomer du kan opleve ved at stoppe med PPI? |
| (Delt) beslutningstagning | Hvor involveret vil du gerne være i beslutningen?  Hvordan tænker du at beslutningen skal foregå? | Er det vigtigt at der tages hensyn til dine preferencer?  Hvem synes du er ansvarlig for at tage den endelige beslutning? (Dig selv, lægen eller en helt tredje) |
| Praktiske forhold | Hvad har du brug for af din læge, for at du tænker at det kan lade sig gøre at stoppe med PPI?  Andet end rådgivning fra din læge, hvad tænker du ellers du har brug for, for at tage sådan en beslutning? | Har du brug for andre end din læges og din mening til at træffe en beslutning? (Hvem og hvorfor?)  Er det vigtigt for dig at dine pårørende er involveret? Hvis ja, på hvilken måde?  Hvordan vil du have det med skrevet eller visuel information som supplement til diskussionen? (fx i forhold til gevinster ved PPI-stop, risiko ved fortsat PPI-brug osv.)  Hvis ovenstående er for åbent:  Vil det hjælpe din beslutning at have noget skrevet eller visuel information som supplement til diskussionen? (Hvorfor/hvorfor ikke?)  Har du brug for løbende konsultationer for at høre hvordan det går/følge op? |
| Risiko | Vil du have nogen bekymringer i forhold til hvis du skulle stoppe med PPI? (Hvis ja, hvilke?)  Hvordan tænker du, at du vil bringe diskussionen, omkring dine bekymringer, op med din læge? | Hvordan ville du have det med at forsøge at stoppe med PPI, hvis din læge forsikrer at du altid vil kunne vende tilbage til din nuværende behandling, hvis det ikke går? |
| Planlægning | Vil du have brug for at diskutere en plan med din læge for hvordan du skal stoppe din PPI? | Hvad skal denne plan indeholde? |
| Yderligere tanker | Er der andre ting, som er vigtige for dig, når du diskuterer med din læge om din PPI skal stoppes? |  |
